# Metabolic reprogramming in cervical cancer and metabolomics perspectives

**DOI:** 10.1186/s12986-021-00615-7

**Published:** 2021-10-19

**Authors:** Boning Li, Long Sui

**Affiliations:** 1 Obstetrics and Gynecology Hospital, Fudan University, Shanghai, 200011 China; 2 Obstetrics and Gynecology Hospital, Center of Diagnosis and Treatment for Cervical Diseases, stetrics and Gynecology Hospital, Fudan University, Shanghai, 200011 China; 3 Shanghai Key Laboratory of Female Reproductive Endocrine Related Diseases, Shanghai, 200011 China

**Keywords:** Cervical cancer, HPV, p53, Metabolomics, Warburg effect

## Abstract

Cumulative studies have shown that metabolic reprogramming is a hallmark of malignant tumors. The emergence of technological advances, such as omics studies, has strongly contributed to the knowledge of cancer metabolism. Cervical cancer is among the most common cancers in women worldwide. Because cervical cancer is a virus-associated cancer and can exist in a precancerous state for years, investigations targeting the metabolic phenotypes of cervical cancer will enhance our understanding of the interference of viruses on host cells and the progression of cervical carcinogenesis. The purpose of this review was to illustrate metabolic perturbations in cervical cancer, the role that human papillomavirus (HPV) plays in remodeling cervical cell metabolism and recent approaches toward application of metabolomics in cervical disease research. Cervical cancer displays typical cancer metabolic profiles, including glycolytic switching, high lactate levels, lipid accumulation and abnormal kynurenine/tryptophan levels. HPV, at least in part, contributes to these alterations. Furthermore, emerging metabolomics data provide global information on the metabolic traits of cervical diseases and may aid in the discovery of biomarkers for diagnosis and therapy.

## Introduction

Cervical cancer is the fourth most common cancer worldwide, and more than 500,000 new cases of cervical cancer are diagnosed annually with the number of deaths reaching 300,000 every year [1]. Human papillomavirus (HPV) infection is detected in 90% of cervical cancers. High-risk HPV (HR-HPV) is capable of inducing anogenital cancer. Of all the HR-HPV types, HPV16 (50–60%) and HPV18 (20%) are predominately found in cervical cancer cases [2]. A small fraction of individuals who develop persistent infection for over 2 years may develop squamous precursor lesions, which are divided into stage cervical intraepithelial neoplasia (CIN) 1–3 or low-grade/high-grade squamous intraepithelial lesions (LSILs/HSILs) [3]. A minority of high-grade lesions transform into invasive cancer if no intervention occurs [4].

Cervical cancer mainly metastasizes via lymphatic vessels and direct extension. Radical hysterectomy, radiotherapy and cisplatin-based chemotherapy are primary treatment options, yet specific schemes should be carefully considered according to cancer stage [5]. However, more than 30% of patients are radioresistant, and the recurrence of early-stage cervical cancer may be greater than 5% within 4.5 years [6, 7]. However, the current standard-of-care radiation therapy for locally advanced cervical cancer has not been improved for more than 30 years, and clinical outcomes have stagnated. Thus, the development of new treatment strategies is needed to enhance the therapeutic outcome [8, 9]. Several recent studies have indicated that metabolic reprogramming may effectively support cervical cancer therapy by increasing chemo- and radiosensitivity [10].

As early as the 1920s, Otto Warburg first observed that glucose consumption and lactate production in tumors are higher than those in normal tissues. This phenomenon is named the “Warburg effect”, which describes a metabolic feature of cancer in that cancer cells tend to gain energy by glycolysis instead of oxidative phosphorylation, even in the presence of oxygen. Currently, with an increasing number of studies expanding our understanding of cancer-associated metabolic alterations, reprogramming of metabolism has been considered a hallmark of cancer. In their elegant review in 2015, Pavlova and Thompson organized cancer-associated metabolic changes into six hallmarks [11]. Most cancers display several of these hallmarks, but different cancer types and even subtypes of certain cancers show variant metabolic phenotypes. An increasing number of studies are focusing on the metabolic traits of cervical cancer cells, providing novel indications for diagnosis and treatment.

In this literature review, we present recent advances in high-throughput technology to assess the metabolic characteristics of cervical cancer and related diseases. We then discuss metabolic changes and their impact on malignancy in cervical cancer as well as the potential mechanisms behind the alterations.

### Metabolic alterations in cancers: to survive and thrive

An essential issue for cancer cells is to overcome environmental restriction and sustain rapid proliferation. Consequently, cancer cells rewire their metabolism to meet the need to survive and thrive. An increasing number of studies have described and explored the metabolic adaptations of cancer cells. Here, we summarize some of the most studied hallmarks of tumorigenesis-associated metabolic reprogramming.

Active aerobic glycolysis, namely, the “Warburg effect”, is commonly observed in cancers [12] and may be a strategy to gain advantages in the case of limited energy resources. At the same time, glycolytic intermediates support other biosynthesis processes, such as de novo serine synthesis and de novo lipid synthesis [13]. Moreover, the Warburg effect rebuilds the tumor microenvironment to facilitate cancer growth and immune evasion. Secreted lactate contributes to anti-inflammatory M2 tumor-associated macrophage polarization and inhibits tumor surveillance by T cells and natural killer (NK) cells [14, 15].

Lipid metabolism is markedly altered in rapidly proliferating cells. In cancer cells, fatty acids (FAs) are utilized in large part to synthesize sphingolipids and glycerophospholipids to produce cell membrane and signaling molecules. While most normal cells prefer exogenous FA for biosynthesis, de novo FA synthesis is greatly increased in cancer cells [16]. Blocking FA synthesis has been verified to be effective in limiting cancer growth in experimental animal models and may be a potential strategy in future clinical applications [17, 18]. In addition, some cancer cells exhibit increased fatty acid oxidation (FAO, also known as β-oxidation) activity, and FAs are mainly catabolized by FAO. FAO is required for the maintenance and chemoresistance of cancer stem cells, and it prevents detached cancer cells from anoikis, thus improving metastasis [19, 20].

Alterations in amino acids are increasingly being recognized. In mammalian cells, glutamine metabolism provides carbon for the biosynthesis of amino acids and FAs, and it provides nitrogen for nucleotide synthesis. Glutamine also functions in numerous biological processes, including bioenergetics, antioxidative defense mechanisms and cell signaling regulation [21]. Many types of cancer cells show increased consumption and dependence on glutamine, and some cancer-promoting pathways boost glutamine metabolism. For example, MYC upregulation increases glutamine metabolism enzymes and transporters; protein kinase Cζ (PKCζ) loss stimulates glutamine metabolism [22].

In addition to the features mentioned above, other features, including active nucleotide metabolism and other complex metabolic alterations, are also widely observed in cancers. In general, cancer cells promote biosynthesis pathways to produce cellular components and utilize multiple energy sources.

### Current status of metabolomics in cervical disease research: potential tool for identifying diagnostic markers and therapeutic targets

Metabolomics is a field of omics science that has followed genomics, transcriptomics and proteomics. Metabolomics aims to analyze the metabolic profile of cells, body fluids or tissues, enabling the identification of diverse metabolites and exploration of intrinsic biological mechanisms. High-throughput, high-resolution and high-sensitivity technology has been extensively used in various areas, including human science, animal science, drug discovery, microbiology, plant biology, food chemistry and environmental science [23, 24]. Based on analytical chemistry techniques, the 3 leading platforms of metabolomics are nuclear magnetic resonance (NMR), gas chromatography mass spectrometry (GC–MS) and liquid chromatography mass spectrometry (LC–MS) [25]. Several studies have utilized metabolomics as a tool to investigate cervical HPV-related diseases, ranging from infection to lesion to invasive cancer. These descriptive studies have demonstrated evident metabolic alterations during the cervical malignancy process, indicating the significance of exploring the phenotypes, mechanism and impact of cervical cancer metabolic traits. The results are summarized in Table 1.

**Table 1:** Metabolomic analyses in cervical lesions and cervical cancer

Year	Group and sample size	Sample origin	Method	Major findings	References
2008	5 normal cervix, 45 CIN, 23 cervical cancer	Biopsy specimens	HR-MAS MRS	1. **Choline and phosphocholine** increased in cancer compared with high-grade CIN tissue 2. **Phosphoethanolamine** was increased in cancer compared with normal tissue 3. **Alanine and creatine** were reduced in normal tissue from cancer patients compared with normal tissue from non-cancer patients 4. **Choline** was increased in CIN tissue from cancer patients compared with CIN tissue from non-cancer patients 5. **Choline-containing metabolites** increased from pre-invasive to invasive cervical cancer	[22]
2017	Training set: 70 cervical cancer, 80 normal control; testing set: 66 cervical cancer, 69 normal control	Plasma	LC–MS	1. 55 metabolites were down-regulated in cervical cancer patients while 7 metabolites were up-regulated 2. **Bilirubin, LysoPC(17:0), *n*-oleoyl threonine, 12-hydroxydodecanoic acid and tetracosahexaenoic acid** can be biomarkers for cervical cancer diagnosis	[23]
2018	40 normal cervix, 40 HSIL	Cervical cytologic specimens	LC–MS	2 **ceramides** and 1 **sphingosine** metabolite are unique signatures for HSIL, and occurred independently of HPV status	[24]
2018	42 negative for intraepithelial lesion or malignancy (NILM), 34 SIL	Plasma	Electrospray ionization coupled to Q Exactive Orbitrap MS (lipidomics)	**Prostaglandins, phospholipids, sphingolipids, Tetranor-PGFM and hydroperoxide lipid** are distinct lipids to identify NILM and SIL	[25]
2019	69 normal, 55 CIN1, 42 CIN2/3, 60 cervical cancer	Plasma	LC–MS	1. **AMP, aspartate, glutamate, hypoxanthine, lactate, proline, and pyroglutamate** were discriminated between CINs and cervical cancer versus normal 2. The seven metabolites combined with positive HPV status were correlated with substantial risk of cancer progression	[26]
2019	18 healthy HPV−, 11 healthy HPV+, 12 LSIL, 27 HSIL, 10 cervical cancer	Cervicovaginal lavages and vaginal swabs	LC–MS	1. **Three-hydroxybutyrate, eicosenoate, and oleate/vaccenate** discriminated between cancer patients versus healthy 2. ICC group had an enrichment of **amino acid metabolites** in comparison to other groups that were HPV positive (healthy HPV+, LSIL, and HSIL) 3. **Lipid, xenobiotics, and carbohydrate super-pathways metabolites** enriched in the ICC group compared to other groups	[27]
2019	13 HPV-negative, 26 HPV-positive (including 14 HR-HPV)	Self-collected mid-vaginal swabs	LC–MS	HPV+ women had higher **biogenic amine** and **phospholipid** concentrations compared with HPV– women after adjustment for vaginal microbiota Community State Type and cigarette smoking	[28]
2021	66 healthy controls, 55 CIN1, 44 CIN 2/3, 60 cervical cancer	Plasma	Ultraperformance liquid chromatography/quadrupole time-of-flight MS (UPLC-QTOF-MS, lipidomics)	The levels of most **diglyceride and FFA** species were higher, while the levels of most **phosphatidylcholine and phosphatidylethanolamine** species were lower in the patients with CIN 2/3 and cervical cancer than in the healthy controls and the patients with CIN1	[31]

Summary of the design and findings of studies that applied metabolomics to investigate metabolic features in cervical diseases (HPV infection, cervical lesions and cervical cancer). The studies are listed in chronological order

The specific metabolites included in *Major findings* are shown in bold texts

Similar to other types of studies on cancer, many metabolomics studies focus on differences between cancer and normal tissues. Yang et al. tested plasma samples from cervical cancer patients and normal controls with LC–MS-based untargeted metabolomics. In cervical cancer patients, Yang and colleagues reported that 55 metabolites were downregulated and that 7 metabolites were upregulated, and they suggested that the combination of bilirubin, lysophosphatidylcholine (17:0) (lysoPC(17:0)), *n*-oleoyl threonine, 12-hydroxydodecanoic acid and tetracosahexaenoic acid are satisfactory candidate biomarkers for cervical cancer diagnosis [26]. Ilhan and colleagues collected cervicovaginal lavages from HPV-negative controls, HPV-positive controls and women with cervical dysplasia or cancer, and they reported that cervical cancer exhibits an abundance of metabolites in lipid, xenobiotic and carbohydrate pathways in comparison to other groups. Ilhan et al. indicated that oleate/vaccenate, eicosenoate and 3-hydroxybutyrate are good indicators to discriminate cervical cancer patients from healthy controls [27].

The total course from HPV infection to invasive cervical cancer can take up to 20 years. Early diagnosis and clinical intervention of HSILs is of great importance because HSILs are identified as precancerous lesions. Using LC–MS analysis of cervical cytologic specimens, Porcari and colleagues identified 3 unique metabolites classifying HSIL and normal cervix independent of HPV status, including a sphingosine metabolite and two ceramides [28].

Because cervical cancer is often a virus-related disease, metabolic alterations might be attributed to both HPV infection and cancer progression. The HPV infection impact has also been studied using metabolomics. Analysis of samples from self-collected mid-vaginal swabs has indicated that HPV+ women have higher biogenic amine and phospholipid concentrations than HPV− women [29].

Using lipidomics, a subfield of metabolomics, Neves et al. demonstrated that 5 types of lipids, including prostaglandins, phospholipids, sphingolipids, tetranor-prostaglandin F and its metabolite (dihydroketoprostaglandin; tetranor-PGFM), as well as hydroperoxide contribute to the distinction of blood plasma samples from individuals who have no intraepithelial lesions or malignancies from those who have squamous intraepithelial lesions. The diversity of plasma lipids in healthy individuals and cervical dysplasia indicates metabolic modulation in cervical malignancy progression [30]. In a lipidomics study profiling the global lipids of plasma, Nam et al. showed that phosphatidylcholine, phosphatidylethanolamine, diglyceride and free FAs are major lipid classes with significant differences in patients with CIN2/3 and cervical cancer compared to healthy controls and patients with CIN1 [31]. However, these studies did not determine the same specific metabolites to identify cervical lesion progression. Although the findings of metabolomics are comparable to wet-lab studies, it is difficult to target a specific metabolite of cervical cancer through the articles we summarized in this research. However, discrepancies in these studies might be due to diversities in instruments, algorithms, classifications of metabolites and grouping methods of patients.

The design of a metabolomics study is based on the purpose of research. Using high-resolution magic angle spinning magnetic resonance spectroscopy (HR-MAS MRS), Silva et al. showed increased choline and phosphocholine in cervical cancer tissue compared to high-grade CIN tissue, and they reported that the evels of alanine and creatine are lower in normal tissue from cancer patients than in normal tissue from healthy individuals [32]. Because this study used in situ tissue and covered the entire process from a normal state to dysplasia and to cancer, it helped to investigate the transition from preinvasive to invasive disease. However, Khan and colleagues used LC–MS to study plasma samples from CIN patients, cervical cancer patients and normal controls, and they reported that the top 7 metabolites differing among the normal, CIN and cancer groups are adenosine monophosphate (AMP), aspartate, glutamine, hypoxanthine, lactate, proline and pyroglutamate. These combined 7 metabolites reveal satisfactory diagnostic value with an area under the curve (AUC) of 0.83 between the normal and cervical cancer groups, indicating that high levels of targeted metabolites with positive HPV status show a significant risk for the development of CIN2/3 and cervical cancer [33]. Due to the thorough AUC analysis and combination with HPV status, the study by Khan and colleagues is valuable for identifying biomarkers of cervical cancer.

Current metabolomics studies in cervical diseases are restricted to descriptive research of clinical samples, and few experiments are performed in these studies to confirm the transformation of metabolites and enzymes. Nevertheless, high-throughput technology would be beneficial to identify nonmainstream, alternative metabolic features in cervical diseases and inspire in-depth molecular mechanism research.

### HPV plays a role in metabolic regulation: how viruses contribute to carcinogenesis

HPV is a double-stranded DNA virus, and the genome of HPV is divided into 8 open reading frames (ORFs), coding viral early genes E1, E2, E4, E5, E6 and E7 as well as late genes L1 and L2 [34, 35].

Viruses depend on the availability of host metabolic constituents to replicate and accomplish their life cycles. Therefore, viruses evolve to manipulate metabolism in infected host cells for better adaptation [36]. Evidence from high-throughput technologies supports that HPV infection is implicated in cell metabolism modulation. Zheng and colleagues investigated the global circRNA expression profiles in E7-transfected Ca Ski cells using bioinformatics analysis; GO enrichment analysis showed that the upregulated circRNAs are enriched in “oxidative phosphorylation” but that the downregulated circRNAs are enriched in “polyamine metabolic process”, “regulation of protein catabolic process” and “neutral amino acid transport”; while Kyoto Encyclopedia of Genes and Genomes (KEGG) pathway analysis indicated that the upregulated circRNAs are enriched in “FA metabolism” but that the downregulated circRNAs are enriched in “arginine and proline metabolism”, “glutathione metabolism” and “central carbon metabolism in cancer” [37]. In 3-dimensional organotypic raft cultures of cervical tissues, Kang and colleagues performed a GO enrichment analysis, revealing that early HPV16 infection-induced downregulated genes are enriched in “sphingolipid biosynthetic process” and “unsaturated FA metabolic process” [38]. In this section, we will introduce verified and speculated mechanisms of how HPV promotes metabolic reprogramming in cervical epithelial cells (Table 2).

**Table 2:** Association between HPV and metabolic alterations

HPV viral protein	Direct target	Secondary target	Enzyme	Metabolic process
E6	c-MYC		HK2	Glycolysis
	miR-143-3p		HK2	Glycolysis
	p53	miR-34a	LDHA	Lactate
				Glycolysis
			SREBP1C	Lipogenic
			Lpin1, MCD, CPT1	FAO
		miRNA-107, PANK1	FASN, SCD1, CPT1α	Lipid metabolism
			SLC7A3	Arginine uptake
			GLS2	Glutamine metabolism
E7	miR-143-3p		HK2	Glycolysis
	Rb1		GLUT1, HK2, PKM2	Glycolysis
		E2F1	GLUT2, GCK, PDK4, FBP, PKLR	Glycolysis
			ACACA FASN, CHREBP, SREBP1C, CKB,	Lipogenic
	PI3K/Akt/mTOR		PFK1, GLUT	Glycolysis

Summary of the mechanisms by which HPV regulates metabolism. The responsible HPV viral proteins, their targets, the modified enzymes and corresponding metabolic processes are listed

#### HPV remodels glucose metabolism

Glycolytic flux is the best-described metabolic alteration induced by HPV infection. HPV E6 O-GlcNAcylates and stabilizes c-MYC [39], which enhances the transcription of hexokinase 2 (*HK2*), the rate-limiting enzyme responsible for the first step in glycolysis [39, 40]. Furthermore, E6/E7 induces downregulation of the *HK2*-inhibitory microRNA, miR-143-3p, thereby promoting glycolysis [41]. E6 inactivates p53, which directly suppresses miR-34a expression. Reduction of miR-34a promotes the Warburg effect and elevates the level of lactate dehydrogenase A (LDHA), which is responsible for the processing of pyruvate to lactate [42, 43]. Thus, lactate production is increased in both the intracellular and conditioned media of HPV-positive cells [44]. In addition, amplification of the phosphatidylinositol 3-kinase (PI3K)/Akt/mammalian target of rapamycin (mTOR) signaling cascade is frequent in HPV-induced cancers [45]. Akt-dependent changes are closely associated with aerobic glycolysis. Hyperactivation of Akt upregulates the expression and membrane translocation of glucose transporters, reverses forkhead box O (FoxO)-induced glycolytic gene suppression and activates key glycolytic enzymes, such as phosphofructokinase 1 (PFK1) [46].

#### The E6 and E7 viral proteins potentially impact host cell metabolism

The E6 and E7 oncogenes in HR-HPV types are closely related to malignant transformation of host cells. E6 and E7 interact with the p53 and pRb tumor suppressors, respectively. As a result, p53 is degraded, thus blocking its proapoptotic effect. pRb disassociates from and consequently activates the E2 factor (E2F) transcription factor, which upregulates cell cycle-related genes. Thus, hyperactivated E6 and E7 drive infected cervical cells into S phase, leading to uncontrolled cell proliferation [47, 48]. Consistent expression of E6 and E7 is required for cervical cancer cell malignant phenotype maintenance.

The metabolic modulations by HPV infection may also be driven by E6- and E7-induced inhibition of p53 and Rb activity. p53 is a well-established tumor suppressor and lipogenic inhibitor that inhibits sterol-regulatory element binding protein 1c (SREBP1C) [49]. Activated p53 directly induces transcription of FA metabolism regulator lipid phosphate phosphohydrolase 1 (Lpin1) and malonyl-CoA decarboxylase (MCD), which catalyzes the degradation of malonyl-CoA to acetyl-CoA and enhances carnitine palmitoyltransferase 1 (CPT1). As a result, p53 increases long-chain FA uptake by the mitochondria, thereby promoting FAO [50, 51]. p53 also binds to the promoter of pantothenate kinase 1 (*PANK1*) to increase the expression of PANK1 and its intronic miRNA-107. PANK1 and miRNA-107 enhance lipid metabolism by upregulating key enzymes involved in lipid metabolism, including fatty acid synthase (FASN), stearoyl-CoA desaturase 1 (SCD1) and CPT1α [52]. In contrast, p53 deficiency facilitates lipid accumulation [53]. Therefore, perturbation of p53 might rebuild lipid homeostasis in HPV-infected cells. Moreover, p53 modulates intracellular amino acid levels. p53 activation leads to transcriptional upregulation of arginine transporter human cationic amino acid transporter 3 (SLC7A3) to promote arginine uptake [54]. Glutaminase 2 (*GLS2*), encoding a key enzyme in the conversion of glutamine to glutamate, is also a target gene of p53. Elevated GLS2 increases glutamate and α-ketoglutarate, which, in turn, enhances mitochondrial respiration and adenosine triphosphate (ATP) generation [55, 56].

Conroy reported that in Kras-driven lung tumors, loss of *Rb1* increases the expression of the following key glycolytic enzymes: glucose transporter 1 (GLUT1), HK2 and pyruvate kinase type M2 (PKM2). The loss of *Rb1* enhances glycolytic metabolism without altering mitochondrial pyruvate oxidation but has no significant effect on tricarboxylic acid (TCA) anaplerosis or utilization of alternative nutrient sources, including lactate and glutamine [57]. However, whether the pRb/E2F1 axis participates in this process is unclear. E2F1 acts as a transcription factor and associates with promoters of several lipogenic or glycolytic genes, such as *FASN*, carbohydrate response element binding protein (*CHREBP*), *SREBF1C*, creatine kinase B (*CKB*), *PDK4* and fructose-2,6-bisphosphatase (*FBP*). E2F1 also indirectly regulates other metabolism-related genes, such as *GLUT2*, glucokinase (*GCK*), pyruvate kinase L/R (*PKLR*) and acetyl-CoA carboxylase (*ACC1*). Therefore, in terms of lipid metabolism, Rb inactivation and successful E2F1 induction prompt de novo lipid synthesis and facilitate lipid accumulation. In contrast, E2F1 promotes glycolytic metabolism, representing a metabolic switch from oxidative to glycolytic metabolism that responds to stressful conditions [58–62]. However, the mechanism by which HPV regulates glucose, glutamine and lipid metabolism via p53 and E2F1 should be confirmed in cervical epithelial cells and cervical tissue specimens.

The metabolic changes induced by HPV, including activated glycolysis, elevated amino acid uptake and de novo lipid synthesis, are consistent with the findings in various cancers. These similarities highlight the solid connection between HPV and malignant transformation of cervical cancer.

### Metabolic features in cervical cancer and carcinogenesis effects: highly associated with malignant phenotypes

#### Glycolysis

In a previous investigation involving 134 nondiabetic patients with cervical cancer (IIB–IVA), measurement of nonfasting plasma glucose levels before starting chemotherapy or radiation indicated that patients with higher glucose levels over 102 mg/dL have a shorter overall survival and progression-free interval compared to their counterparts. This study indicated that glucose level is associated with poor prognosis of cervical cancer, implying a potential interrelationship between cervical cancer and glucose metabolism [63].

As glucose cannot spread freely through the lipid bilayer cell membrane, GLUTs mediate the intake of glucose, and GLUT1 is a leading carrier for glucose in many cancers [64]. The intensity of GLUT1 in low-grade cervical dysplasia is similar to that in normal tissues but is significantly greater in invasive cancer and increases with cancer progression. The overexpression of GLUT1 may be a late phenomenon in cellular transformation [65, 66]. Regarding cervical cancer, higher GLUT1 expression is positively related to higher tumor stage and pelvic lymph node metastasis. Higher expression of GLUT1 is an independent prognostic factor for the overall survival of HPV16-positive patients, and HPV16-positive patients overexpressing GLUT1 have lower immune cell scores of CD8+ T cells, B cells and Th1 cells [67]. GLUT1 expression correlates with HPV infection [68]. Several pathways may contribute to the regulation of GLUT1 in cervical cancer. PKM2, which converts phosphoenolpyruvate (PEP) and adenosine diphosphate (ADP) to pyruvate to produce ATP, is a rate-limiting enzyme of glycolysis. PKM2 is abundant in radioresistant patients and is increased after radiotherapy [69]. PKM2 enhances glycolysis and upregulates the expression of GLUT1 [70]. Another mechanism is that programmed cell death ligand 1 (PD-L1) directly binds to integrin β4 (ITGB4), activates the AKT/GSK3β signaling pathway and consequently promotes snail homolog 1 (SNAI1) expression. SNAI1, which is a transcription factor, negatively regulates sirtuin 3 (SIRT3) by inhibiting its promoter activity. SIRT3 plays an important role in the regulation of glucose metabolism by upregulating GLUT1 and GLUT4 as well as the glycolytic enzymes, HK2 and LDHA, ultimately promoting glucose uptake and glycolysis [71].

6-Phosphofructo-2-kinase/fructose-2,6-biphosphatase 3 enzyme (PFKFB3) and FBP are two glycolysis regulators upregulated in chemoresistant cervical cancer cells, and they increase glycolytic levels (exhibiting high glucose uptake, high ATP levels and low oxygen consumption rates) in cervical cancer cells [72]. Phosphatidylinositol 3-kinase p110 a (*PIK3CA*) E542K and E545K mutants are common in cervical cancer. Mutants also activate the AKT/GSK3β signaling pathway to promote the expression and nuclear accumulation of β-catenin, subsequently suppressing SIRT3 to stimulate glycolysis [73].

Inhibition of glycolysis impairs cervical cancer cell proliferation, indicating the vital role of glycolysis in cervical cancer development [39]. Abnormal glucose metabolism also contributes to chemoresistance. Active glycolysis activates hypoxia inducible factor-1α (HIF-1α), mediates autophagy and reduces chemosensitivity [74].

#### Lactate

The miR-34a/LDHA axis facilitates cervical cancer cell proliferation and invasion [43]. The plasma lactate concentration is lowest in individuals without cervical lesions and increases according to cervical lesion grades, reaching a maximum in cervical cancer. Lactate produced by cervical cancer cells stimulates the secretion of interleukin-6 (IL-6) and IL-10 as well as upregulates HIF-1α expression and decreases p65 NF-κB activity in tumor-associated macrophages, contributing to a procancerous M2 macrophage phenotype. Modulated macrophages are less potent in T cell activation, promoting a protumoral microenvironment [75]. Lactate inhibits histone deacetylases (HDACs) and induces histone H3 and H4 hyperacetylation as well as decreases chromatin compactness to create a DNA repair-proficient environment. Furthermore, lactate induces the expression of genes involved in DNA repair, such as the homologous recombination-related gene, Nijmengen breakage syndrome 1 (*NBS1*), and the nonhomologous end joining (NHEJ) genes, DNA ligase 4 (*LIG4*) and aprataxin (*APTX*), while enhancing the activation and nuclear accumulation of DNA-dependent protein kinase (DNA-PKcs), a key enzyme involved in NHEJ. Multiple mechanisms cooperate in DNA repair and improve cell viability after chemotherapy [76].

#### Lipids

Lipid metabolism is altered in cervical cancer. Cumulative evidence indicates that lipid droplets gradually increase in noncancerous cervical tissue, preinvasive dysplastic cervical epithelium and invasive cervical cancer [77, 78]. Moreover, chemoresistant cervical cancer cells contain more lipid droplets than chemosensitive cells [72]. Regulation of lipid enzymes is involved in this process. Long noncoding RNA LNMICC is increased in cervical cancer with lymph metastasis and poor prognosis. LNMICC activates FA metabolism by upregulating several key FA metabolic enzymes, including FASN, ACC1, ACOX1, CPT1α and FABP5, which consequently upregulates intracellular triglycerides and phospholipids [71].

Lipids comprise nearly 50% of the cell membrane. Distinct membrane lipid composition in cervical cancer cells may be an indication of altered lipid metabolism. Compared to their nonmalignant counterparts, cervical cancer cells undergo a loss of cell membrane rigidity due to the following changes: the level of sphingomyelin 16:0 decreases; the proportion of phospholipids with shorter fatty acyl chain lengths increases; and FAs are more desaturated [79]. The changes in lipid compositions facilitate cell signaling, and a less rigid membrane may help cancer cells move the vascular wall forward to promote migration [80, 81]. The adjustment in membrane lipids may be attributed to HPV because HPV 16 E5 increases the synthesis rate of phosphatidylcholine and phosphatidylserine but diminishes the synthesis rate of phosphatidylglycerol in keratinocytes [82]. Some lipids regulate the cell membrane to facilitate their own uptake, which indirectly promotes cancer malignancy. Oleic acid elevates the membrane expression of CD36, a FA transporter, and induces Srs kinase, activating the downstream ERK1/2 pathway to promote cervical cancer cell proliferation and invasion in a CD36-dependent manner [62].

#### Amino acids

GLS, which catalyzes the conversion of glutamine to glutamate, is positively related to glutamine metabolism. GLS2 is enhanced in radioresistant cervical cancer and produces antioxidants, including glutathione (GSH), NADH and NADPH, to suppress reactive oxygen species (ROS) levels in response to radiation [83]. Similarly, in chemoresistant cervical cancer, loss of miR-497 leads to upregulation of transketolase, resulting in increased glucose uptake and GSH generation, which reduces ROS and chemosensitivity [84].

**Fig. 1:**
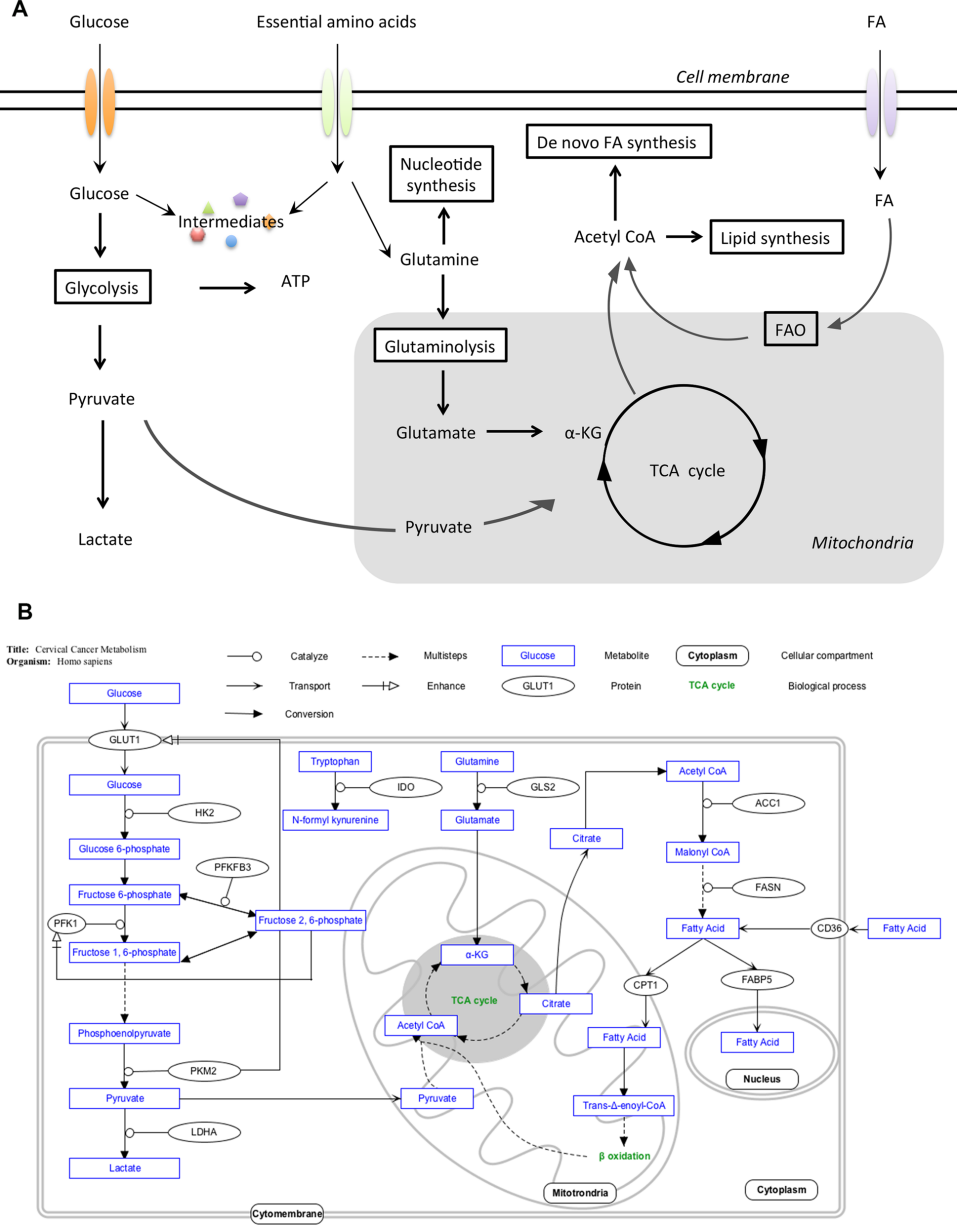
Metabolic regulation in cancer and cervical cancer. **A** Various cancers share several metabolic features in common. Here, we briefly illustrate typical characteristics of cancer metabolism. Generally, the uptake of glucose, amino acids and FAs is increased. Glycolysis is the leading energy source and consequently increases lactate production. Intermediates of glycolysis and glutamine metabolism are used for various biosynthesis pathways. De novo synthesis of FA is increased, and the generated FAs are further utilized in signal transduction, cellular component constitution and FAO. **B** Glycolysis, FA metabolism and amino acid metabolism are the most affected pathways in cervical cancer. The enzymes and transporters in the figure are all enhanced in cervical cancer or positively related to tumor aggressiveness. The figure was generated using PathVisio 3.0.0+ [102]

In mammals, tryptophan is used for protein and indole synthesis. Indoleamine 2,3-dioxygenase (IDO) is the rate-limiting enzyme that catalyzes tryptophan degradation in the kynurenine pathway and acts as an immune checkpoint. In the tumor microenvironment, IDO exhausts tryptophan to produce kynurenine and its derivatives, which are toxic to T cells. As a result, the T cell immune response is suppressed, leading to tumor microenvironment immunologic tolerance [85, 86]. Regardless of HPV infection status, normal cervical squamous epithelial cells have low IDO expression, but in women with SIL or cervical cancer, the percentage of IDO-positive cervical epithelial cells or leukocytes in their cervical tissue increases [87]. Ferns and colleagues demonstrated that cervical cancer patients with tumor bulks greater than 4 cm or lymph node metastatic spread have lower plasma tryptophan levels; these researchers reported that higher kynurenine and 3-hydroxykynurenine concentrations are positively correlated with advanced FIGO stage and lymph node metastases, and they suggested that a high kynurenine/tryptophan ratio is related to poor disease-specific survival [88]. However, cervical tissue samples from the same cohort revealed no difference in survival outcome between IDO-positive and IDO-negative patients. Compared to patients with patchy IDO expression, patients with marginal tumor IDO expression at the interface with the stroma have improved disease-free survival and disease-specific survival [89]. In addition, IDO-positive tumor-infiltrating immune cells are related to fewer lymph node metastases. Different sampling tissues (plasma and cervical tissue) may account for the divergence between these two studies. Hence, tryptophan metabolic traits may differ between blood and tumors (Fig. 1, Table 3).

**Table 3:** Metabolites, enzymes and transporters involved in cervical cancer and their related phenotypes

Metabolic pathway	Specific factors involved		Related phenotypes
Glycometabolism	Metabolites	Lactate	Pro-cancerous M2 macrophage, DNA repair after chemotherapy
	Enzymes	LDHA	Proliferation, invasion
		PKM2	Radioresistant
Lipid metabolism	Metabolites	Membrane compositions	Migration
		Oleic acid	Proliferation, invasion
	Transporters	CD36	
Amino acid metabolism	Metabolites	Kynurenine	Immunologic tolerance, metastasis

Several specific metabolites, enzymes and transporters have been confirmed in experimental studies to be related to cancerous phenotypes in cervical cancer. Metabolic alterations are relevant to various malignant biological properties of cancer, including increased proliferation, distant metastasis and immune escape

### Targeting metabolism in cervical cancer therapy: theoretically possible but requires clinical verification

Because metabolic reprogramming is crucial for cancer malignancy, targeting cancer metabolism might be a competent strategy to support conventional cancer therapy. Glucose metabolism modulators are the most widely studied, and related drug discovery has made significant progress. Several TCA regulators have been applied to clinical practice. Enasidenib (IDHIFA; AG-221), an IDH2 inhibitor, was approved by the US Food and Drug Administration (FDA) in 2017 for the treatment of adult patients with mutant *IDH2* relapsed or refractory acute myeloid leukemia (R/R AML) [90]. The IDH1 inhibitor, ivosidenib (TIBSOVO, AG-120), was approved by the FDA in 2018 to treat mutant-*IDH1* R/R AML. In a multicenter, randomized, double-blind, placebo-controlled, phase 3 study by ClarIDHy, ivosidenib also significantly improved the progression-free survival of advanced *IDH1*-mutant cholangiocarcinoma patients without severe intolerance [91]. Several drugs with other metabolic regulator capacities have been involved in clinical trials. After favorable safety results in a phase 1 study cohort, the GLS1 inhibitor, CB-839, has been combined with cabozantinib in the CANTATA study, an ongoing international, randomized, double-blind, multicenter study, to evaluate its efficacy in inhibiting metastatic renal cell carcinoma [92]. In the open-label phase 1/2 study, ECHO-202/KEYNOTE-037, epacadostat, an IDO1 selective inhibitor, and the PD-1 inhibitor, pembrolizumab, have been well tolerated and associated with promising responses in nonsmall cell lung cancer (NSCLC) and melanoma patients [93, 94]. However, a further phase 3 trial demonstrated that the combination of epacadostat and pembrolizumab is not superior to the combination of placebo and pembrolizumab in improving survival outcomes in patients with unresectable or metastatic melanoma [95]. Phase 3 clinical trials in other cancers are still ongoing [96]. The uncertainty of the results of the usefulness of metabolic regulation in cancer therapy strategies may be due to the following reasons: cultured cells have difficulty completely simulating cancer metabolism in the tumor microenvironment; there are compensation pathways for metabolic inhibition; and there is metabolic heterogeneity in cancer cells although cancer cells are metabolically distinct from adjacent normal counterparts [97, 98].

Some studies have illustrated potent metabolic targets for cervical cancer treatment. Caffeic acid downregulates GLS and malic enzyme 1 (ME1), which suppresses pyruvate dehydrogenase kinase (PDK) activity, thereby disturbing the TCA cycle. Caffeic acid also impairs de novo synthesis of unsaturated FA. Metformin inhibits glycolytic enzymes, including GLUT1, GLUT3, HK2, PFKFB4, PKM and LDH. The combined application of caffeic acid and metformin disrupts energetic homeostasis, restrains proliferation and promotes apoptosis in cervical cancer cells. Moreover, caffeic acid and metformin increases the sensitivity to cisplatin chemotherapy [99, 100]. Inhibition of glycolysis, glutathione metabolism or thioredoxin metabolism radiosensitizes cervical cancer cell lines and xenografts [101]. These results indicate that metabolic intervention may be a prospective adjuvant treatment to support anti-cervical cancer therapy. Because the related studies are mainly confined to in vitro and animal experiments, further investigation should be performed to determine whether metabolic adjuvants have potential in clinical application.

## Conclusions

Metabolic adaption is a recognized feature of cancer. Many cancer types share the following similar metabolic traits: glycolysis is active even in oxygen-rich environments; de novo FA synthesis and FAO are increased; and glutamine consumption greatly increases. However, metabolic heterogeneity occurs in different cancer types. Cervical cancer remains a major threat to women’s health. High-throughput omics technology has been applied to analyze biopsy tissue, cytological specimens or vaginal lavage fluid, revealing metabolic divergence between normal cervical tissues and lesions, between uninfected and HPV-infected cells or between benign tissues and tumors. Metabolomics may contribute to further mechanistic investigation and precise cervical cancer diagnosis. In addition to metabolic phenotypes, the initiation of cervical cancer metabolic alterations has been investigated in a number of studies. Because persistent HR-HPV infection is the key to cervical epithelial cell transformation, the role of HPV viral proteins in metabolic modulation has been investigated. Many studies have demonstrated that HPV is related to remodeling of glucose metabolism and that it may also participate in lipid and glutamine regulation. Metabolic alterations benefit cervical cancer growth and several processes, including chemoresistance, radioresistance, invasion and immune escape.

Nevertheless, unresolved questions remain in this field. The utility of metabolomics opens a new field to discover potential biomarkers for cervical cancer and cervical lesion diagnosis. However, some changes revealed by metabolomics are not yet well understood. It may be worthwhile to explore why these alterations occur and how the altered metabolites affect cervical cancer progression. Previous studies on cervical cancer metabolism have suggested that metabolism may be a target for cervical cancer therapy. Although progress has been achieved using in vitro and animal experiments, evidence from clinical studies is still lacking. Future mechanistic studies and rigorously designed clinical studies will move the field forward.

## Data Availability

Not applicable.

## Abbreviations

HPV: Human papillomavirus
HR-HPV: High-risk HPV
CIN: Cervical intraepithelial neoplasia
LSIL: Low-grade squamous intraepithelial lesion
HSIL: High-grade squamous intraepithelial lesion
NK: Natural killer
FA: Fatty acid
FAO: Fatty acid oxidation
PKCζ: Protein kinase Cζ
NMR: Nuclear magnetic resonance
GC–MS: Gas chromatography mass spectrometry
LC–MS: Liquid chromatography mass spectrometry
lysoPC: Lysophosphatidylcholine
PGFM: Prostaglandin F and its metabolite
HR-MAS MRS: High-resolution magic angle spinning magnetic resonance spectroscopy
AMP: Adenosine monophosphate
AUC: Area under curve
ORF: Open reading frame
KEGG: Kyoto Encyclopedia of Genes and Genomes
HK2: Hexokinase 2
LDHA: Lactate dehydrogenase A
PI3K: Phosphatidylinositol 3-kinase
mTOR: Mammalian target of rapamycin
FoxO: Orkhead box O
PFK1: Phosphofructokinase 1
SREBP1C: Sterol-regulatory element binding protein 1c
Lpin1: Lipid phosphate phosphohydrolase 1
MCD: Malonyl-CoA decarboxylase
CPT1: Carnitine palmitoyltransferase 1
PANK1: Pantothenate kinase 1
FASN: Fatty acid synthase
SCD1: Stearoyl-CoA desaturase 1
SLC7A3: Arginine transporter human cationic amino acid transporter 3
GLS2: Glutaminase 2
GLUT1: Glucose transporters 1
PKM2: Pyruvate kinase type M2
TCA: Tricarboxylic acid
E2F: E2 factor
CHREBP: Carbohydrate response element binding protein
CKB: Creatine kinase B
FBP: Fructose-2, 6-bisphosphatase
GCK: Glucokinase
PKLR: Pyruvate kinase L/R
ACC1: Acetyl-CoA carboxylase
ATP: Adenosine triphosphate
PEP: Phosphoenolpyruvate
PD-L1: Programmed cell death ligand 1
ITGB4: Integrin β4
SNAI1: Snail homolog 1
SIRT3: Sirtuin 3
PFKFB3: 6-Phosphofructo-2-kinase/fructose-2, 6-biphosphatase 3 enzyme
PIK3CA: Phosphatidylinositol 3-kinase p110 a
HIF-1α: Hypoxia inducible factor-1 α
IL-6: Interleukin-6
HDAC: Histone deacetylase
NBS1: Nijmengen breakage syndrome 1
NHEJ: Non-homologous end joining
LIG4: DNA ligase 4
APTX: Aprataxin
DNA-PKcs: DNA-dependent protein kinase
ROS: Reactive oxygen species
IDO: Indoleamine 2,3-dioxygenase
NSCLC: Non-small cell lung cancer
ME1: Malic enzyme 1
PDK: Pyruvate dehydrogenase kinase
